# A Case Report on Endometriosis-Associated Intestinal Tumour Following Hysterectomy: Diagnostic Challenges and Implications for Hormone Replacement Therapy (HRT) Guidelines

**DOI:** 10.7759/cureus.74306

**Published:** 2024-11-23

**Authors:** Sara Elsaadany, Ashweta Josan, Ruth Crowley, Michelle Gouldie

**Affiliations:** 1 General Practice, Barking, Havering and Redbridge Primary Care, London, GBR; 2 Emergency Medicine, Barking, Havering and Redbridge University Hospitals National Health Service (NHS) Trust, London, GBR; 3 Internal Medicine, Barking, Havering and Redbridge University Hospitals National Health Service (NHS) Trust, London, GBR

**Keywords:** bowel endometriosis, endometrial associated intestinal tumour, hyper-oestrogenism, merkel cell carcinoma of unknown primary, oestrogen-only hormone replacement therapy, total abdominal hysterectomy with bilateral salpingo-oophorectomy (tahbso)

## Abstract

Endometriosis is a complex condition that is often underdiagnosed, leaving rare but serious risks such as malignant transformation overlooked in many cases. Malignant transformation, especially in extra-gynaecological sites, poses significant diagnostic and therapeutic challenges. We present a case of an endometriosis-associated intestinal tumour diagnosed in a patient with exposure to oestrogen-only hormone replacement therapy (HRT) on a background of total abdominal hysterectomy and bilateral oophorectomy performed three decades before her presentation. This case highlights the potential for malignant transformation of residual endometriotic tissue in extra-gynaecological sites, even decades following definitive surgical management, particularly in the presence of important risk factors such as obesity and prior oestrogen-only HRT.

## Introduction

Endometriosis is defined as the extrauterine presence of endometrial tissue. It affects 10% of reproductive-aged women, making it the second most common gynaecological disorder [1]. Despite clear protocols, it remains a significant challenge to diagnose and manage, with uncertainties regarding the epidemiology and pathogenesis. Current National Institute for Health and Care Excellence guidelines state that definitive investigations, such as laparoscopy, are only performed if symptoms persist or worsen despite treatment, contributing to diagnostic delays that average around eight years in the United Kingdom [2,3]. These diagnostic delays are more pronounced in deep infiltrating endometriosis, as the involvement of non-gynaecological pathways complicates the diagnostic process. This delay, in combination with frustrations from repeated missed diagnoses and poorly controlled symptoms, has a significant impact on the quality of life of women [4].

Malignant transformation of endometriosis is rare, with an estimated incidence of 1%, ovarian cancer being the most prevalent and extensively studied form; 21.3% of these cases occur at extragonadal pelvic sites, with endometriosis-associated intestinal tumours (EAITs) being more uncommon [5].

We present a case of a woman who, due to her diagnosis of endometriosis, had undergone total abdominal hysterectomy and bilateral oophorectomy 30 years before her diagnosis with an EAIT. This case highlights the alarming possibility of malignant transformation of residual endometriotic tissue in distant sites, even decades after what was thought to be a definitive surgery.

## Case presentation

A 69-year-old woman (gravida 2, para 2 and body mass index, BMI, 40) presented to primary care with a four-week history of fatigue, unintentional weight loss, left-sided cramping abdominal pain and altered bowel habits marked by increased frequency of loose stools. Her past medical history was notable for suspected endometriosis with ovarian endometrioma, for which she underwent a total abdominal hysterectomy and bilateral oophorectomy at 39 years old. Other comorbidities included type 2 diabetes mellitus, managed with basal-bolus insulin, and hypertension, for which she was on a regimen of beta-blockers, alpha-blockers and thiazide diuretics. She also has a solitary kidney with JJ stents due to a previous nephrectomy secondary to obstructive uropathy. Histopathological examination following her hysterectomy confirmed endometriosis with ovarian involvement. Postoperatively, she received oestrogen-only hormone replacement therapy (HRT) intermittently over 13 months. There was no significant family history of colorectal or endometrial cancer.

Diagnostic assessment

Given the patient's presentation, a comprehensive blood panel was performed, revealing anaemia and persistently elevated inflammatory markers (Table 1). In light of these findings, alongside her symptoms of weight loss, altered bowel habits and abdominal pain, an urgent referral was made under the two-week wait pathway to investigate for lower gastrointestinal malignancy.

**Table 1:** Initial blood test results of the patient, which warranted onward referral under the two-week wait pathway

Blood tests	Previous results	Results after presentation	Normal ranges
Haemoglobin	115 g/L	105 g/L	115-155 g/L
White blood cells	11.6 × 10^9^ g/L	13.3 × 10^9 ^g/L	3.8-11 × 10^9^ g/L
Mean corpuscular volume	86.1 fL	79.4 fL	90-96 fL
Platelet count	338 × 10^9^ g/L	531 × 10^9^ g/L	150-400 × 10^9^ g/L
C-reactive protein	29 mg/L	34 mg/L	0-5 mg/L

A colonoscopy was subsequently conducted, revealing a large, circumferential, fungating mass located 32 cm from the anal verge in the sigmoid colon. The lesion appeared friable, exhibited significant vascularity, and was highly suspicious of malignancy, causing near-complete obstruction of the bowel lumen. Multiple biopsies were taken from the mass for histopathological examination. The case was promptly referred for discussion at the colorectal multidisciplinary team (MDT) meeting to determine the appropriate diagnostic and therapeutic course of action.

**Figure 1:**
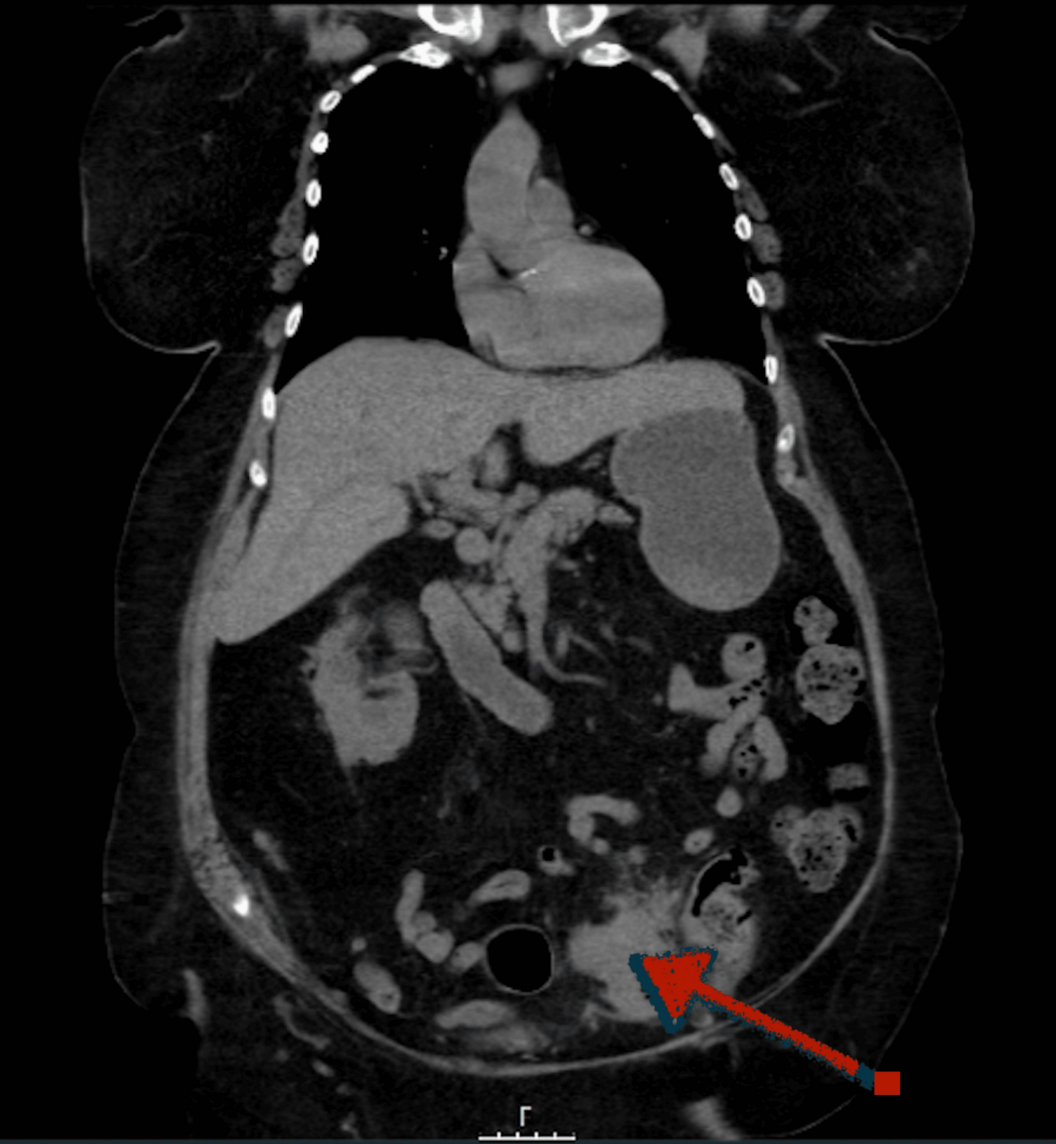
Coronal section of the CT chest-abdomen-pelvis, showing sigmoidal mass nearly obstructing the bowel (red arrow) CT: computed tomography

**Figure 2:**
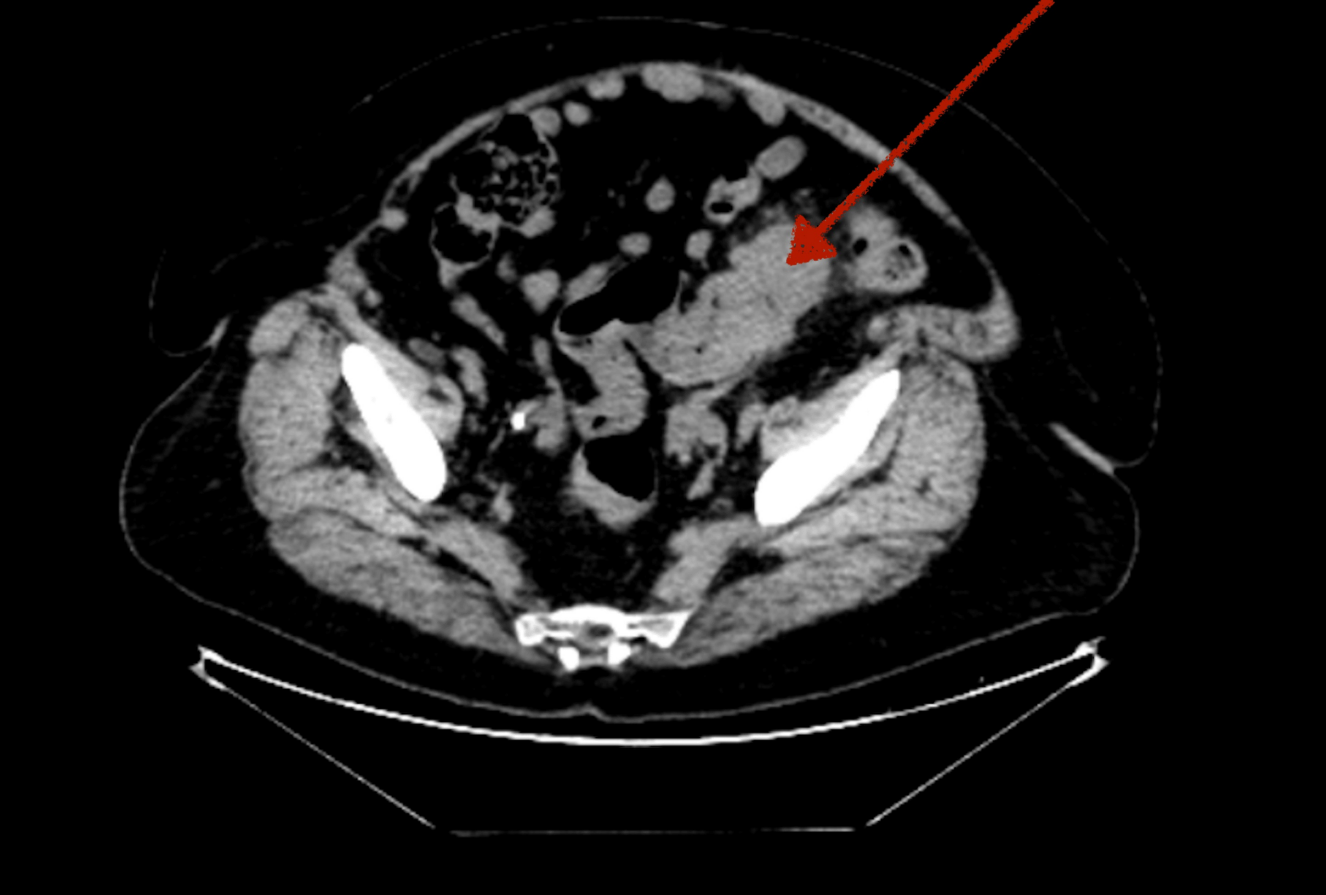
Axial section of the CT chest-abdomen-pelvis, showing the sigmoid mass (red arrow) CT: computed tomography

Computed tomography (CT) imaging of the chest, abdomen and pelvis identified the sigmoid tumour with associated peritoneal involvement, along with small and indeterminate lung nodules (Figures 1, 2). Follow-up positron-emission tomography confirmed metabolic activity in the primary sigmoid mass, consistent with N1 nodal involvement, and no evidence of distant metastasis, leading to a staging classification of T4N1M0. Additionally, CA125 levels were assessed and found to be within normal limits.

Histopathological examination, including immunohistochemical staining, revealed necrotic fragments of adenocarcinoma extending through the entire bowel wall, from the mucosa through the muscularis propria and into the serosal fat. Adjacent tissue showed foci of endometriosis. The immunohistochemical profile demonstrated positivity for cytokeratin 7, vimentin and CD10, along with oestrogen receptor positivity, further characterising the tumour as of endometrial origin.

Management

Following MDT review, the patient underwent an open laparotomy, including sigmoid colectomy, appendectomy, small bowel resection and a boari flap reconstruction due to extensive endometriosis affecting the right kidney. Histopathological analysis confirmed an endometrial origin for the malignancy, with clear surgical margins achieved. The case was subsequently presented at the gynaecology-oncology MDT, where it was recommended that the patient begin adjuvant chemotherapy with a regimen of six cycles of carboplatin and paclitaxel.

**Figure 3:**
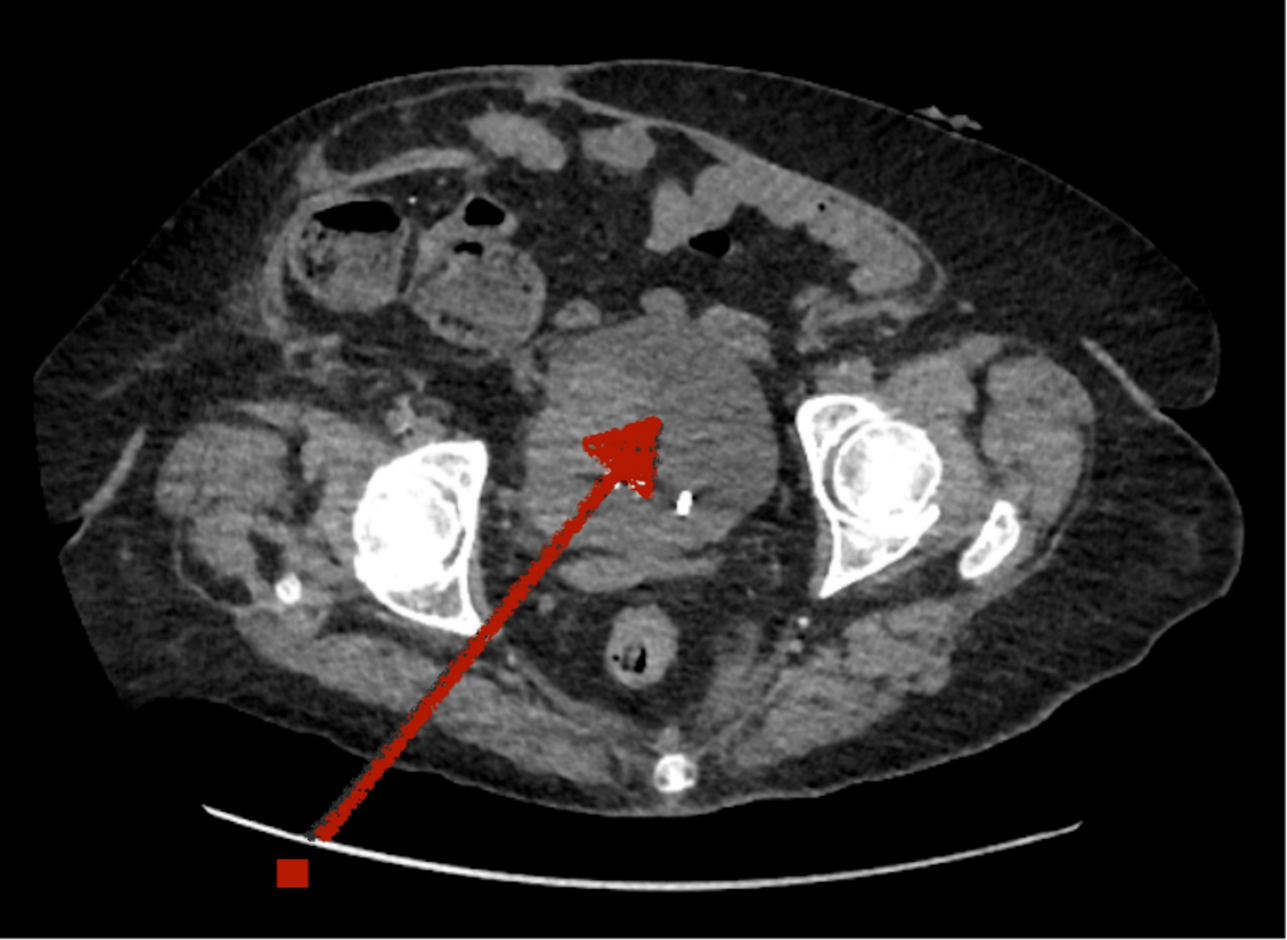
CT urinary tract, showing suspected bladder invasion (red arrow) CT: computed tomography

The patient remained in remission for three years, with regular blood tests and CT scans showing no signs of recurrence. However, a CT of the urinary tract to investigate haematuria incidentally revealed new hepatic lesions and possible bladder invasion (Figure 3) from adjacent peritoneal soft tissue disease, which were not present on imaging four months earlier. Ultrasound-guided liver biopsy with immunohistochemical staining suggested a likely Merkel cell carcinoma origin for these lesions. Unfortunately, the patient subsequently experienced a prolonged hospital stay due to multi-organ failure and passed away shortly after that.

## Discussion

EAITs usually present with symptoms misdiagnosed as irritable bowel syndrome or, less commonly, as intestinal cancer due to the overlapping presenting symptoms. Given the ambiguity of the patient's presentation and her risk stratification for gynaecological malignancy being low due to her total abdominal hysterectomy with bilateral oophorectomy, she was referred under the lower gastrointestinal malignancy pathway for suspected intestinal cancer [6].

There have been around 50 reported cases of EAIT, out of which 72% have involved the recto-sigmoid colon, with a large proportion being adenocarcinoma. Presentation is usually between the late 20s and early 50s, occurring much earlier than the usual incidence of intestinal carcinomas. Furthermore, after the discovery of the intestinal tumour, differentiation between EAIT and primary colon malignancy can prove challenging. Due to its rare occurrence, there are no guidelines on the therapeutic approach for EAIT, which usually requires an MDT approach [6].

This reflects the diagnostic and management process the patient experienced. She presented with non-specific symptoms with a mass that appeared malignant on colonoscopy. The case was discussed in the colorectal MDT, and the decision was to treat it as sigmoid cancer, pending the immunohistochemical results. Despite gynaecology input and immunohistochemical results, management remained under the guidance of the colorectal team until post-surgical confirmation of the endometrial origin of the cancer.

Post-hysterectomy, the patient was commenced on intermittent oestrogen-only HRT for 13 months. Hyper-oestrogenism, whether from an endogenous or exogenous source, is a known risk factor for the malignant transformation of endometriosis.

With regard to this case, risk factors for endometrial cancer included prolonged exposure to unopposed oestrogen in the context of underlying endometriosis and obesity [7]. Obesity, a known cause of hyper-oestrogenism, is increasing in prevalence. Studies show a statistically significant association between patients with a higher BMI who were also taking unopposed oestrogens and developing a malignant transformation of endometriosis [8].

Unopposed oestrogen therapy is becoming more common due to more women being commenced on HRT. Given the increasing incidence of obesity as well, we can presume that there could be an increasing prevalence of cancer developing from endometriosis in the future [8].

A systematic review highlights the link between HRT and malignant transformation of endometriosis post-menopause. This study concludes that key patient characteristics, including previous endometriosis and definitive gynaecological surgery prior to menopause resulting in prolonged oestrogen-only HRT, were seen in the majority of case studies [9]. Most up-to-date guidelines consider this risk by recommending combined HRT for patients with confirmed endometriosis who have undergone total hysterectomy [10].

Further guidelines are needed to determine if women already receiving progesterone via an intrauterine system for the treatment of endometriosis require additional supplementation when starting HRT to ensure sufficient progesterone dosing. A tailored, risk-based HRT with regular monitoring, even long after surgical interventions, is essential to improve outcomes for both confirmed and suspected cases of endometriosis.

## Conclusions

The case highlights the complexity and long-term implications of endometriosis, a benign condition with the rare potential for malignant transformation. A significant, unexpected complication showcased here is the potential for malignant transformation of residual endometriotic tissue in extra-gynaecologic sites, even decades following definitive surgical management.

Current guidelines have now changed to recommend combined HRT to mitigate risks for patients with confirmed endometriosis who have undergone hysterectomies. However, gaps remain for patients with suspected but unconfirmed endometriosis who have undergone hysterectomies for other reasons. This case report stresses the need for updated guidelines to close this essential gap for women.
